# Xeno-Free In Vitro Cultivation and Osteogenic Differentiation of hAD-MSCs on Resorbable 3D Printed RESOMER^®^

**DOI:** 10.3390/ma13153399

**Published:** 2020-07-31

**Authors:** Marline Kirsch, Annabelle-Christin Herder, Cécile Boudot, Andreas Karau, Jessica Rach, Wiebke Handke, Axel Seltsam, Thomas Scheper, Antonina Lavrentieva

**Affiliations:** 1 Institute of Technical Chemistry, Leibniz University Hannover, Callinstraße 5, 30167 Hannover, Germany; kirsch@iftc.uni-hannover.de (M.K.); annabelle.herder@gmail.com (A.-C.H.); scheper@iftc.uni-hannover.de (T.S.); 2 Evonik Nutrition & Care GmbH, Business Line Health Care, Kirschenallee, 64293 Darmstadt, Germany; cecile.boudot@evonik.com (C.B.); andreas.karau@evonik.com (A.K.); 3 German Red Cross Blood Service NSTOB, Institute Springe, Eldagsener Straße 38, 31830 Springe, Germany; Jessica.Rach@bsd-nstob.de; 4 Bavarian Red Cross Blood Service, Institute Nuremberg, Heimerichstrasse 57, 90419 Nuremberg, Germany; w.handke@blutspendedienst.com (W.H.); a.seltsam@blutspendedienst.com (A.S.)

**Keywords:** resorbable polymers, 3D printing, in vitro biocompatibility, RESOMER^®^, in vitro degradation, osteogenic differentiation, human platelet lysate, human serum, fetal calve serum, adipose tissue-derived mesenchymal stem cells (hAD-MSCs)

## Abstract

The development of alloplastic resorbable materials can revolutionize the field of implantation technology in regenerative medicine. Additional opportunities to colonize the three-dimensionally (3D) printed constructs with the patient’s own cells prior to implantation can improve the regeneration process but requires optimization of cultivation protocols. Human platelet lysate (hPL) has already proven to be a suitable replacement for fetal calf serum (FCS) in 2D and 3D cell cultures. In this study, we investigated the in vitro biocompatibility of the printed RESOMER^®^ Filament LG D1.75 materials as well as the osteogenic differentiation of human mesenchymal stem cells (hMSCs) cultivated on 3D printed constructs under the influence of different medium supplements (FCS, human serum (HS) and hPL). Additionally, the in vitro degradation of the material was studied over six months. We demonstrated that LG D1.75 is biocompatible and has no in vitro cytotoxic effects on hMSCs. Furthermore, hMSCs grown on the constructs could be differentiated into osteoblasts, especially supported by supplementation with hPL. Over six months under physiological in vitro conditions, a distinct degradation was observed, which, however, had no influence on the biocompatibility of the material. Thus, the overall suitability of the material LG D1.75 to produce 3D printed, resorbable bone implants and the promising use of hPL in the xeno-free cultivation of human MSCs on such implants for autologous transplantation have been demonstrated.

## 1. Introduction

Additive manufacturing, or three-dimensional (3D) printing has become an important and promising part of regenerative medicine and tissue engineering in recent years. To date, over 2500 publications on the topic of 3D printing in tissue engineering are published in PubMed, with over 90 percent in the past five years. 3D printing had its early start at the beginning of the 1980s and since that time gained popularity in increasing number of application fields [1,2,3]. Besides the application in food industry, military, engineering, and automobile industry, the use of 3D printing in regenerative medicine and tissue engineering increasingly gains the attention of researchers [1,2,3,4,5].

One of the widely used 3D printing techniques in bioregenerative medicine is the fused deposition modeling (FDM) of synthetic polymers [2]. In this process, material is applied via an extruder and printing head onto a platform. By this common and inexpensive layer by layer process, a wide range of materials can be precisely printed [2,6,7,8,9]. Depending on the application, a specific polymer can be used to precisely adjust hydrophobicity, degradation time, stability, and mechanical properties [8,9,10,11]. One of the greatest advantages is reproducible production, which successfully offers inexpensive replacement products for donor organs [2]. 3D printed scaffolds can maintain the desired cell phenotypes and morphology, stimulate the formation of functional tissue, and promote differentiation [7,12,13]. In several studies the positive impact of the application of 3D scaffolds on tissue regeneration has already been proven [12].

In addition to already extensively studied permanent implant materials, such as ceramics, composites and metal alloys, biodegradable polymers can also be applied [14,15]. The use of alloplastic biodegradable materials provides many advantages for regenerative medicine. The fact that 3D printed constructs degrade in the body after fulfilling their purpose prevents a second surgical intervention and thus improves the patient’s quality of life. The risk of post-surgical infections is also subsequently reduced [15,16]. The optimal in vivo degradation rate would correspond to the tissue regeneration rate, so that the degradable implant remains in the patient’s body only until the body’s own tissues have regenerated themselves [17,18]. By creation of personalized tissue constructs, tissue engineering with 3D printed resorbable polymers has the potential to increase quality and length of life [10]. Particularly in oral and maxillofacial surgery, a wide variety of studies have already demonstrated the advantages of using resorbable biomaterials [19,20,21,22,23,24]. For instance, 3D printing can optimize surgery as well as rehabilitation time and facilitate pre- and intraoperative decisions which improves the efficacy and the accuracy of surgeries [20]. Especially in infants, progressive growth would require a second surgery to remove the permanent implant [11]. This surgery can be avoided by using resorbable implants.

To ensure successful implantation of 3D printed biodegradable constructs in the human body, the used material must be biocompatible through the entire process of biodegradation in the body so that no toxic degradation compounds are released into the body [25]. Therefore, the printing process must not trigger the formation and release of toxic substances and not alter the original material properties such as resorbability and mechanical stability [15]. In combination with the process of 3D printing, the developed resorbable materials can be used to manufacture highly personalized implants adapted to the demands of each patient [11]. This can be achieved by converting the results of medical imaging techniques such as X-rays, computer, or magnetic resonance tomography into 3D objects [26,27,28,29]. A more detailed design can lead to an improved regeneration dynamics and treatment outcome. For example, Hsu et al. demonstrated that a larger inner space of FDM-generated scaffolds could stimulate osteoblast cell growth, a highly porous and interconnecting structure could promote cell ingrowth and a concentric structure may be conducive for osteoblast growth [8].

Considering the broad field of applications of biodegradable implant materials, the question of the application-specific suitable material becomes crucial. One of the most widely used biocompatible materials in the field of tissue engineering is poly(lactic-co-glycolic acid) (PLGA) [30,31,32,33,34,35,36]. This biocompatible copolymer is characterized by good mechanical properties such as firmness, excellent processability, and the degradation time can be individually adapted to the respective use via the lactic acid (LA), glycolic acid (GA) ratio. Guo et al. investigated the effects of ester and acid end caps as well as the effect of different LA:GA ratios in PLGA [7].

They demonstrated that a high LA:GA ratio and an ester cap can provide a higher structural stability of the polymer during the printing process and the scaffold structure and mechanical strength can be maintained over a relatively long period of time [7,37].

As promising implant polymer, PLGA degrades primarily by chemical hydrolysis of the hydrolytically unstable ester bonds. The generated products such as lactic acid and glycolic acid can be broken down and excreted via the common metabolic pathways [33,38]. In addition to the implantation of printed alloplastic resorbable constructs into the human body, it is also possible to colonize the 3D printed constructs with the patient’s own cells such as mesenchymal stem cells (MSCs) prior to implantation to additionally support the healing process [39]. Increasingly, xeno-free cultivation conditions of MSCs are being intensively studied in order to avoid the use of animal-derived supplements, such as fetal calf serum (FCS). Several advantages of human platelet lysate (hPL) as a valuable alternative for FCS have already been demonstrated, such as its human origin, its easy accessibility, good manufacturing practice (GMP) conform production capability and a wide range of bioactive factors. Furthermore, hPL has already shown positive results in 2D and 3D cell cultivation because of excellent expansion and differentiation support of MSCs [40,41,42]. Furthermore, Jonsdottir-Buch et al. showed no difference between using expired or fresh platelet concentrates for the preparation of hPL as medium supplement. Therefore, the combination of two promising and sustainable approaches, such as cultivation in hPL and creation of degradable 3D printed constructs could be a highly favorable strategy for regenerative medicine.

In the present work, we investigated the suitability of 3D printed constructs made from the resorbable PLGA filament LG D1.75 (Evonik Industries AG, Essen, Germany) with a LA:GA ratio of 85:15 for use as biomaterial for implant manufacturing with regard to its general in vitro cytotoxicity and biocompatibility to human MSCs. Furthermore, the in vitro degradation behavior of the constructs was investigated and its influence on the biocompatibility of the material was examined. Additionally, a comparative study of proliferation and differentiation of MSCs on the printed constructs under the influence of three supplements (FCS, HS, hPL) was performed.

## 2. Materials and Methods

### 2.1. Printing and Sterilisation of the Resorbable Constructs

The used RESOMER^®^ Filament LG D1.75 (85:15) is an amorphous copolymer consisting of L-lactide and glycolide filament provided by Evonik Industries AG (Essen, Germany). By using FDM printing with a nozzle temperature of 230–250 °C (3NTR, Oleggio, Italy) 1 cm^2^ constructs of the RESOMER^®^ with a filament diameter of 1.75 mm were printed as a 45° crosshatch with three layers (1 mm). The chamber temperature was always lower than 35 °C. The average filament size of the 3D printed constructs was determined microscopically and measured 424.6 ± 36.95 µm. Pictures of four different constructs were taken and the width of three filaments on each picture were measured (twelve-fold determination) (Figure S1, Supplementary Materials). After the printing process the constructs were sterilized for 30 min in 70% ethanol at 37 °C followed by washing with sterile phosphate buffer saline (PBS) [15]. Until further use the constructs were stored in the freezer at −20 °C to prevent spontaneous degradation.

### 2.2. Cell Cultivation

Human adipose tissue-derived mesenchymal stem cells (hAD-MSCs) were isolated from adipose tissue after abdominoplastic surgery. All patients gave their informed consent, which was approved by the Institutional Review Board (Hannover Medical School) with the reference number 3475-2017. In order to identify the isolated cells as mesenchymal stem cells, the cells were examined for several characteristics as already described in a previous study [43]. The as hAD-MSCs-characterized cells were expanded in alpha-MEM medium (Thermo Fisher Scientific, Waltham, MA, USA) supplemented with 10% human serum (HS, CC-pro, Oberdorla, Germany), and 0.5% gentamicin (Merck Millipore, Darmstadt, Germany). The cells were passaged by using accutase (Merck KGaA, Darmstadt, Germany) and cryopreserved in alpha-MEM medium with 20% HS, 0.5% gentamycin, and 10% dimethyl sulfoxide (DMSO) until the start of the experiment. After revitalization the cells were cultivated at 37 °C in a humidified atmosphere of 5% CO_2_ in air. The experiments were performed with cells of passages 2–10 only.

### 2.3. In Vitro Biocompatibility of LG D1.75

#### 2.3.1. In Vitro Toxicity of Extracts

According to ISO 10993-12:2012 [44], each construct was placed in proliferation medium (alpha-MEM medium containing 10% HS and 50 μg/mL gentamicin) with extraction ratio of 3 cm^2^/mL (surface of printed construct area/media volume) [15]. Together with a control (medium without construct) it was incubated at 37 °C for 72 ± 1 h. hAD-MSCs were plated in 96-well plates with cell densities of 8000 cells/well. After 24 h, cells were treated with extract dilutions for 24 h at 37 °C. Afterwards, the cell viability was determined by CellTiter-Blue^®^ Assay (CTB, Promega, Mannheim, Germany) (incubation time of 90 min). Cells treated with medium incubated without printed constructs were used as control and considered as 100% of cell viability.

#### 2.3.2. Direct Cell Growth on Constructs

The sterilized and washed constructs were placed in the middle of a 24-well plate and 8000 cells per well were seeded. As control the same cell number per square centimeter was seeded in wells without constructs. After one day, the constructs were moved in a new well plate, where the cells grew only on the surface of the construct. After one, three, and seven days of cultivation the viability of the cells was evaluated by CTB assay (Promega, Mannheim, Germany) and calcein-acetoxymethyl (AM, Merck, Darmstadt, Germany) and ethidium-homodimer (Sigma Aldrich, München, Germany) staining. CTB assay was performed according to the information provided by the manufacturer. Briefly, a 10% CTB solution was prepared with α-MEM (1:10 v/v) and 500 µL were added to each well after carefully aspirating the old medium. Additionally, wells without cells were filled with the 10% CTB solution and used as a blank. The fluorescence was measured with a microplate reader (Fluoroskan Ascent, Thermo Fisher Scientific Inc., Waltham, MA, USA) at an extinction wavelength of 544 nm and an emission wavelength of 590 nm after 4 h of incubation at 37 °C. To each well 500 µL of a staining solution with 4 µM calcein-AM, 4 µM ethidium-homodimer in α-MEM were added and incubated for 45 min at 37 °C and evaluated with a Cytation 5-Cell Imaging Multi-Mode Reader (Biotek Instruments, Winooski, VT, USA).

### 2.4. In Vitro Degradation Study

The in vitro degradation of the 3D printed RESOMER^®^ Filament LG D1.75 constructs was performed in sterile Kirkland’s Biocorrosion Medium (KBM: 5.4 g/L NaCl, 2.2 g/L NaHCO_3_, 0.9 g/L D-Glucose, 0.38 g/L KCl, 0.28 g/L CaCl_2_, 0.122 g/L Na_2_HPO_4_ (Anhydrous), 0.06 g/L MgSO_4_). Therefore, the constructs were incubated for over six months in 3 mL KBM/construct at 37 °C and the medium was exchanged every other week. The degree of degradation was evaluated microscopically as well as gravimetrically and surface changes were analyzed by 3D microscopy (Keyence VHX-6000, Keyence Deutschland GmbH, Neu-Isenburg, Germany). Prior to the start of the experiment, all constructs were weighed and compared with the measurements after 0.5, 1, 2, 3, 4, 5, and 6 months. Constructs were washed with distilled water to remove residual salts, vacuum dried in a desiccator for 24 h at room temperature (RT), weighed and examined for surface degradation on a 3D microscope. Using a 200× magnification, gloss-reduced images of the top of the structure were taken and the entire image area was used for the software analysis with the VHX-5000 Communication Software of the Keyence 3D microscope. A Gaussian filter and end effect correction were used to measure the maximum height (Sz). The degraded constructs were stored at −20 °C until they were sterilized for biocompatibility testing on the degraded surfaces.

### 2.5. In Vitro Biocompatibility of the Partially Degraded Constructs

The biocompatibility test was performed as described previously (Section 2.3). Instead of the new 3D printed constructs, the degraded constructs after 0, 3, 4, 5, and 6 months of in vitro degradation were used to evaluate if the cells can still grow on a degraded surface.

### 2.6. Osteogenic Differentiation on Printed LG D1.75

The hAD-MSCSs were seeded on the 3D printed constructs and cell culture surfaces as control, grown until confluence and differentiated with FCS, HS, or hPL as supplement into osteoblasts. The differentiation medium was composed of 5 mM β-glycerophosphate, 0.2 mM L-ascorbate-2-phosphate, 0.1 µM dexamethasone, 0.5% gentamicin, as well as either 10% FKS, 10% HS, or 2.5% hPL. As a proliferation control, cells were cultured on the construct in proliferation medium. The cells were cultured for 7, 14, or 21 days and the medium was exchanged every 3–4 days. Afterwards they were washed with warm PBS and fixed for 15 min at 4 °C with 4% paraformaldehyde (Merck, Darmstadt, Germany). To evaluate the osteogenic differentiation, the cell culture surface and constructs were stained for 15 min with an alizarin red solution (1% alizarin red S (Merck KGaA, Darmstadt, Germany) in dH_2_O) at RT. The wells were washed with dH_2_O and the red chelates were visualized and examined with a fluorescence microscope (Olympus, IX50, Olympus Corporation, Tokyo, Japan) with a camera (Olympus SC30, IX-TVAD, Olympus Corporation, Tokyo, Japan) and the CellSens Software (CellSens Standard 1.7.1, Olympus Corporation, Tokyo, Japan). To quantify the degree of differentiation, the bonded alizarin red chelate complexes were extracted by incubation for 20 min in 10% hexadecylpyridiniumchloride monohydrat (Sigma Aldrich, St. Louis, WI, USA) in PBS at 37 °C and transferred 100 µL/well in a 96 well plate. The absorption was determined with an Epoch Microplate Photospectrometer (BioTek Instruments, Inc., Winooski, VT, USA) at 550 nm. Dilution series was prepared to relate the absorbance to the alizarin red concentration. The concentration was determined by means of a calibration curve. In addition to the alizarin red staining and quantification, the alkaline phosphatase activity of the cells was monitored with 5-bromo-4-chloro-3-indolyl phosphate (BCIP)/nitro blue tetrazolium (NBT) (SIGMAFAST BCIP^®^/NBT, B5655, Merck, Darmstadt, Germany). The cells were washed with PBS and fixed with 4% PFA seven days after induction of the osteogenic differentiation. Afterwards the constructs were covered with the BCIP/NBT solution, incubated for 30 min at RT, washed with PBS, and were microscopically analyzed (Olympus, IX50, Olympus Corporation, Tokyo, Japan).

### 2.7. Statistical Analysis

Data are represented as mean ± standard deviation for triplicate or multiplicate measurements/counts for each sample. Statistical significance was defined as *p* value of 0.05 or less. Statistics were performed using one-way ANOVA (OriginLab, Northampton, MA, USA).

## 3. Results

### 3.1. In Vitro Biocompatibility of Printed LG D1.75

For the application of LG D1.75 in tissue engineering and bioregenerative medicine to produce 3D printed, resorbable implants, it is important that the copolymer is biocompatible. Apart from the absence of general in vitro cytotoxicity, cells should be able to adhere to the surface of the construct and cell proliferation and viability on the construct must not be affected. In addition, the cells should not undergo any morphological or phenotypic changes [29,39].

**Figure 1:**
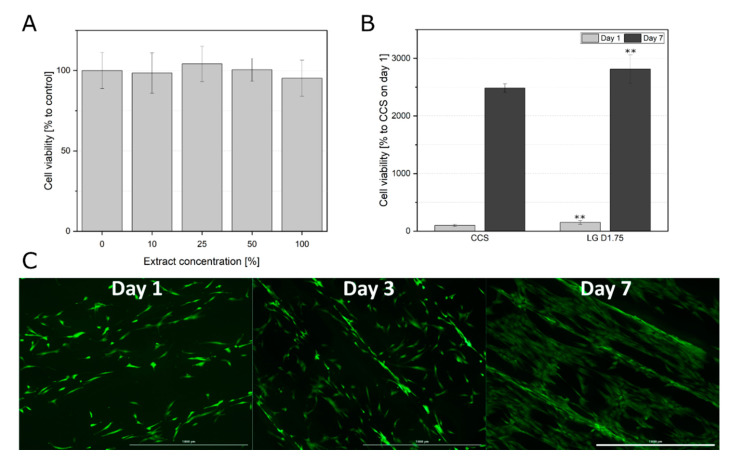
(**A**) Cell viability of hAD-MSCs after 24 h incubation with extraction medium (0, 10, 25, 50, and 100% extraction medium). Data represent the mean ± SD for an eight to ten-fold determination. (**B**) Comparison of cell viabilities of hAD-MSCs grown on the construct surface (LG D1.75) and cell culture surface (CCS) after one and seven days of proliferation. Cell viability determined with the help of CTB assay, CTB fluorescence signal of the cells grown on the CCS on day one served as 100% control. Data represent the mean ± SD for a three to six-fold determination. ** *p* < 0.01. (**C**) Morphological examination of hAD-MSCs growing on the construct surface. After cultivation of 1, 3, and 7 days, the cells were stained with calcein-AM; 4× objective, scale bar 1000 µm.

As first step, 3D printed constructs were tested on possible toxic leachables. After an exposition time of 24 h, the cell viability of the hAD-MSCs cultivated in a dilution series of extracts remained unchanged if compared to the control (cells cultivated in medium without extract) (Figure 1A). Furthermore, the cells were able to adhere to the construct surface and proliferate (Figure 1B,C). Cells demonstrated significantly higher viability on the printed construct, one and seven days after seeding in comparison to the cell culture treated surface in the well plates (Figure 1B). The cells showed an elongated morphology on day 1, although some cells did not fully spread yet (Figure 1C). After one week all cells showed a complete spreading and complex cell network.

### 3.2. In Vitro Degradation

**Figure 2:**
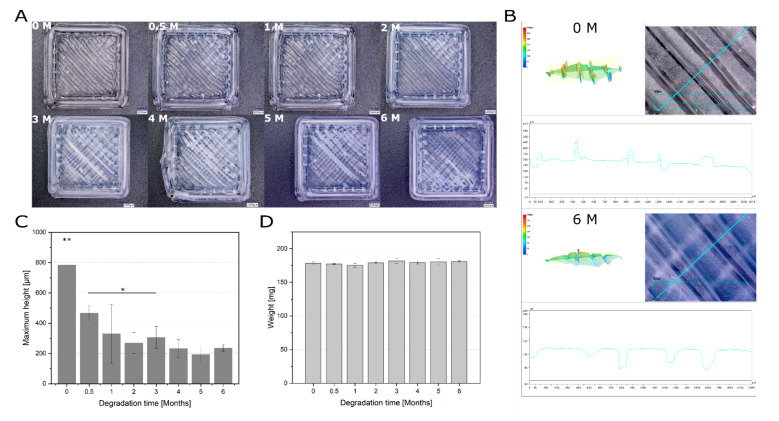
Degradation of printed RESOMER^®^ Filament LG D1.75 over six months. (**A**) Surface of 3D printed constructs before and after different time points of degradation under physiological conditions. Scale bar: 1000 µm. (**B**) Representative 3D profiles of the printed constructs which were used to determine the surface roughness as a threefold determination. One construct without degradation (0 M) and one after six months of degradation (6 M) is shown. (**C**) Maximum height (Sz) as an indicator for the surface roughness of the construct surface during the degradation (200x magnification). Data represent the mean ± SD for a threefold determination. *p < 0.05, **p < 0.01. (**D**) Weight change of printed RESOMER^®^ Filament LG D1.75 during the degradation. Data represent the mean ± SD for a three-fold determination.

As the next step, in vitro degradation rate of 3D printed constructs was evaluated. The in vitro degradation could be detected microscopically over the studied period (Figure 2A). Already after half a month, a slight milky discoloration of the surface could be observed, which was even more evident after the third month. Furthermore, the change in surface roughness during degradation was examined using a 3D microscope by determining the maximum height (Sz). Sz is defined as the sum of the largest peak height value and the largest pit depth value within the defined area. A distinct difference can be observed between the 3D-profile of a construct before the degradation and after six months of degradation (Figure 2B). Under the studied in vitro conditions, the surface roughness decreased significantly over the six months (maximum height before degradation 783.8 ± 150.0 µm and after 6 months of degradation 235.5 ± 20.5 µm) (Figure 2C). This could be examined and confirmed more closely with high magnification (500× and 3000×) micrographs of the construct surface (Figure S2). However, gravimetrically no clear trend could be detected (Figure 2D).

### 3.3. In Vitro Biocompatibility of the Partially Degraded Constructs

**Figure 3:**
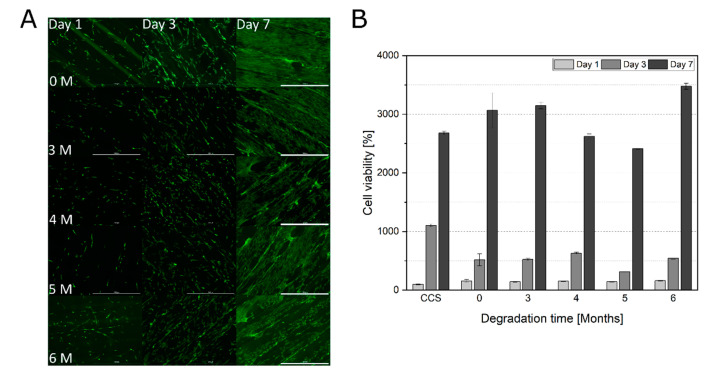
(**A**) Morphological examination of hAD-MSCs growing on degraded RESOMER^®^ PLGA scaffolds (0, 3, 4, 5, and 6 months). After cultivation of 1, 3, and 7 days, the cells were stained with calcein-AM; 4 × objective, scale bar 1000 µm. (**B**) Comparison of cell viabilities of hAD-MSCs grown on degraded RESOMER^®^ PLGA (0, 3, 4, 5, and 6 months) after one, three, and seven days of proliferation. Cell viability determined with the help of CTB assay, CTB fluorescence signal of the cells grown on the CCS on day one served as 100% control. Data represent the mean ± SD for a three-fold determination.

Because the surface properties changed already within the first months of degradation, the in vitro biocompatibility of the degraded constructs after three months was additionally tested. The influence of surface alterations on cell adherence and proliferation was also investigated. Microscopic observations revealed increasing cell spreading from day one to day seven (Figure 3A and Figure S3). Also, cell viability increased over 7 days of cultivation on all studied constructs (Figure 3B). No tendency of changes in morphology, adherence and viability with increasing degradation time could be observed.

### 3.4. Influence of Supplements (FCS, HS and hPL) on Cell Morphology and Osteogenic Differentiation on Printed LG D1.75

#### 3.4.1. Cell Morphology

**Figure 4:**
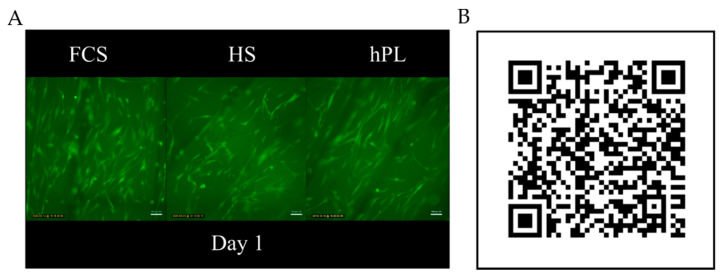
Time-lapse microscope video of hAD-MSCs cultivated on RESOMER^®^ PLGA in medium supplemented with FCS, HS and hPL. (**A**) Single image of the time-lapse video of hAD-MSCs cultivated in medium supplemented with FCS, HS, and hPL on day 1 of cultivation. (**B**) QR code linking to a high speed time-lapse video of hAD-MSCs migrating on RESOMER^®^ PLGA (11270 × speed).

For the application of 3D printed constructs as cell seeded implants in the human body, xeno-free cultivation media is advisable for in vitro cultivation prior implantation. Therefore, the influence of different medium supplements (FCS, HS, hPL) on morphology and cell behavior on 3D printed PLGA constructs was investigated. To observe the cell behavior on the constructs, live time-laps images of the hAD-MSCs growing on the constructs were taken. Independent of the medium supplements, the cells showed a healthy cell spreading and movement on the constructs along the printing direction (Figure 4). Cell morphology and size were nearly the same in all tested supplements.

#### 3.4.2. Osteogenic Differentiation

To investigate whether the LG D1.75 is a suitable material for 3D printed, resorbable bone implants, osteogenic differentiation of hAD-MSCs on the constructs was also investigated. For successful applications, printed materials should not affect cell differentiation. Moreover, as mentioned earlier, the cultivation media plays a crucial role for construct colonization prior to implantation. Thus, the degree of osteogenic differentiation of hAD-MSCs on LG D1.75 was tested comparatively under the influence of FCS, HS, or hPL.

**Figure 5:**
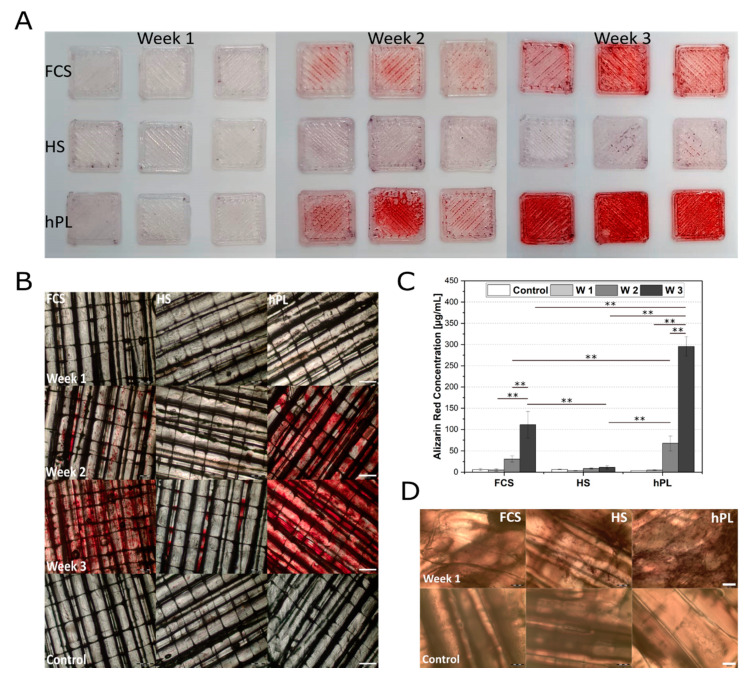
(**A**) Overview over alizarin red stained hAD-MSCs grown on LG D1.75 constructs after 7, 14, and 21 days after induction of the osteogenic differentiation under influence of FCS, HS, and hPL. (**B**) Osteogenic differentiation of hAD-MSCs cultivated on printed RESOMER^®^ Filament LG D1.75 and stained with alizarin red 7, 14, and 21 days after induction of the osteogenic differentiation under the influence of FCS, HS, and hPL. Proliferation control after week 3; 2x objective, scale bar 500 µm. (**C**) Quantification of osteogenic differentiation hAD-MSCs grown on printed RESOMER^®^ Filament LG D1.75 cultivated in medium supplemented with FCS, HS, or hPL. The quantification was performed after day 7, 14, and 21 of differentiation. RESOMER^®^ PLGA in proliferation medium was used as a control. Data represent the mean ± SD for a three-fold (control) and nine-fold (differentiation) determination.** *p* < 0.01. (**D**) Alkaline phosphatase staining of differentiated hAD-MSCs cultivated and for seven days differentiated on printed RESOMER^®^ Filament LG D1.75. Proliferation control after seven days, 4x objective, scale bar 200 µm.

In order to compare the cell behavior on the constructs with the traditional cell culture surface, the osteogenic differentiation of hAD-MSCs was first studied in conventional cell culture plates (Figure S4). Here, hPL supported the osteogenic differentiation of hAD-MSCs significantly better than FCS and HS. This effect could be also observed by cells grown on RESOMER^®^ PLGA (Figure 5A). Only the cells cultured in hPL-supplemented medium showed signs of osteogenesis after one week (Figure S5). After two weeks a clear red coloration of the deposited calcium was detectable, which was even stronger after three weeks indicating an advanced state of mineralization and osteogenic differentiation [42]. For cells cultured in medium supplemented with FCS and HS, first signs of initial osteogenic differentiation could only be detected after two or three weeks, respectively (Figure 5B). The proliferation control showed no spontaneous osteogenic differentiation of hAD-MSCs on LG D1.75 without induction of differentiation (Figure 5B).

For the quantitative determination of the differentiation an alizarin red quantification was performed for the cells growing on LG D1.75 (Figure 5C). The alizarin red quantification showed that hPL could positively influence and significantly support the differentiation of hAD-MSCs on LG D1.75 3D printed constructs. In contrast to hPL (4.7 ± 1.2 µg/mL in week 1 and 295.5 ± 23.0 µg/mL after three weeks), cells differentiated in HS-supplemented medium showed the lowest alizarin red concentrations (8.07 ± 2.77 µg/mL in week 1 and 54.81 µg/mL ± 7.15 after three weeks). In addition to the staining and quantification of alizarin red, cells growing on the constructs were also examined for alkaline phosphatase as an early osteogenic marker. After one week of osteogenic differentiation, hAD-MSCs cultivated in the presence of hPL showed a high number of ALP-positive cells (Figure 5D and Figure S6). In contrast, fewer ALP-positive cells were detected in HS differentiated cells. FCS as supplement led only to single ALP-positive cells.

**Figure 6:**
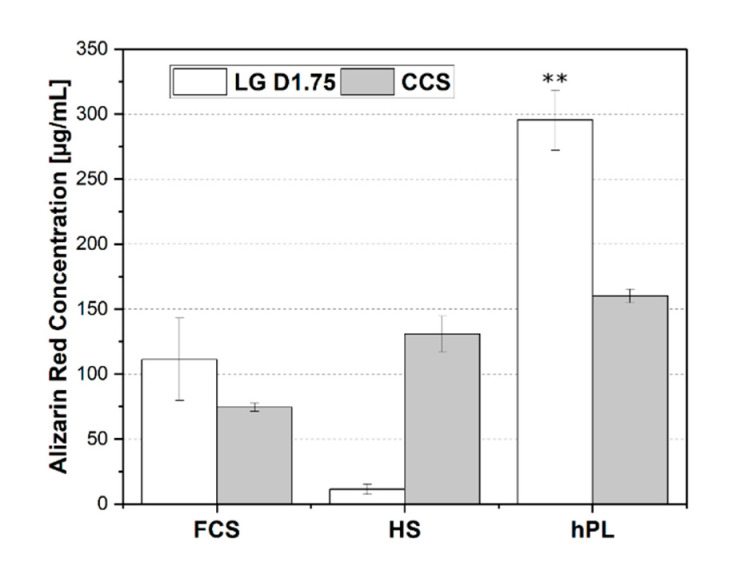
Comparison of alizarin red stained hAD-MSCs grown and differentiated on the printed RESOMER^®^ Filament LG D1.75 construct and on cell culture surface (CCS) for three weeks after induction of osteogenic differentiation under the influence of FCS, HS, and hPL. Data represent the mean ± SD for a three-fold (cell culture surface) and nine-fold (construct surface) determination. ** *p* < 0.01.

In order to compare the differentiation levels of cells grown on RESOMER^®^ PLGA and on the cell culture surface, the quantification of the alizarin red concentration after week 3 was also performed (Figure 6). hPL was more supportive of osteogenic differentiation of hAD-MSCs on the 3D printed constructs after three weeks than on the cell culture surface. The degree of osteogenic differentiation of the hAD-MSCs cultivated with hPL was almost doubled (295.5 ± 23.0 µg/mL) compared to the 2D growing hAD-MSCs on the cell culture surface (160.1 ± 5.2 µg/mL). In contrast, the cells on the constructs using HS medium could hardly differentiate compared to the cell culture surface.

## 4. Discussion

Development of new 3D printable materials for implants provides the possibility to design personalized medical products tailored for each patient according to individual requirements, even for complex medical treatments. 3D printing technology allows the flexibility of single piece manufacturing of the personalized implants of a precise and complex geometry [15,26,27,28]. Besides higher flexibility, application of 3D printing can reduce the production costs. Resorbable polymers represent a novel promising material for implantable medical products, especially preferable in cases where the implant replacement or removal is required. After implantation, these materials also will be reabsorbed in the patient‘s body in a controlled manner, by adjusting the degradation rates to the regeneration speed [17,33]. Recently, bioresorbable polymer RESOMER^®^ Filament LG D1.75 was fabricated as filament for 3D printing (Evonik Industries AG). This polymer consists of PLGA (85:15) and has a degradation time of one to two years. These filaments can be used in FDM printers, where the filament is heated up to 250 degrees in a printing head and uploaded 3D implant models are printed.

Biocompatibility and controlled biodegradability of 3D printed materials is a key issue for their application in medicine. Since FDM printing requires material melting, it is important to exclude the spontaneous generation of possible toxic products [45]. Moreover, the possible introduction of foreign particles between the layers during the 3D printing process is intensively discussed. This can be a critical point, especially with bioresorbable materials since such particles can be released in the body during degradation. For this reason, it must be shown that the printing process itself does not introduce toxic substances.

First, possible toxic leachables from processed material were studied using solid phase extraction method (SPE, according to the ISO 10993-12:2012 [44]. After 24 h of exposition the cells remained vital, regardless of extract concentration. The obtained extracts did not show a toxic effect on human MSCs. Furthermore, the cell adhesion and proliferation on the constructs were investigated to exclude any morphological or phenotypic changes [29,39]. Cultivating the cells directly on the construct surface did not adversely affect the cell behavior. No morphological changes were detectable. The cells showed a high cell viability and remained adhered on the constructs over the entire cultivation period.

Because of the hydrophobic properties of the surface of PLGA scaffolds, many biocompatible materials have been earlier incorporated into PLGA to mimic the extracellular matrix and to enhance the cell adhesion and growth on PLGA [33,46,47]. Fu et al. showed an enhanced effect on proliferation and adhesion of cells on PLGA/hydroxyapaptite nanofiber scaffolds [46]. Ren et al. demonstrated successful adhesion and cell proliferation by cultivating MSCs directly on porous PLGA (85/15) scaffolds [47]. In this study, even a faster proliferation and a higher metabolic activity of the cells growing on the LG D1.75 constructs were observed compared to MSCs growing on cell culture surface. The reason of such good cell adhesion on the 3D printed constructs must be further investigated. A possible explanation is the formation of rough, cell-supporting surface structure during extrusion of the melted material.

A requirement for the use of RESOMER^®^ Filament LG D1.75 as a degradable implant material in regenerative medicine is the decomposition after a certain time in the recipient’s body without releasing toxic substances and without endangering the patient’s health [16]. The rate of degradation and the associated effects on cell growth have a major impact when used as a bone support material. If the construct would dissolve too early after transplantation, the regeneration and promotion of the patient’s own cells cannot be facilitated [15,33,48]. Nevertheless, it should be resorbed after a certain time since a removal after a completed healing process would require another surgical intervention. Furthermore, if the degradation is too slow, the growth and thus the recovery of the recipient can be negatively affected [49].

In this study, the in vitro degradation of the RESOMER^®^ Filament LG D1.75 (with an indicative in vivo degradation time of one to two years) was observed mainly microscopically and by measuring the surface roughness. No clear development could be detected gravimetrically over six months. However, the method used to determine the degradation time plays an important role in the resulted degradation time. The in vitro degradation of a bulk construct in a physiological buffer is not directly comparable to the degradation time of small granulate or tensile bars in the same buffer and also the porosity as well as the pore size has a significant impact [33,50,51]. However, Ren et al. were able to determine an immediate linear weight loss for PLGA with the same LA:GA ratio used in this study (85/15). In contrast to this study, the medium was not changed, so that the pH shift caused by degradation enhanced the autocatalysis of PLGA [33,47]. In our study, the in vitro degradation was performed in a buffered system with regular buffer change to prevent a pH shift. In addition to employing a different pH range, Ren et al. used 90% porous PLGA cut in 7 mm × 4 mm pieces instead of bulk constructs, which results in an increased degradation of PLGA [47]. Wu et al. examined the weight loss of a porous PLGA (85/15) scaffold with 87 ± 3% porosity and showed 16 weeks of constant weight followed by a dramatic weight loss afterwards [48]. These results support the importance of a controlled pH-value for in vitro degradation experiments and the awareness of the previously demonstrated effect of the material fragment size on the degradation rate [33]. Factors like shape, structure, pH, and temperature can be easily adjusted for an in vitro degradation test for physiological conditions. But even if the in vitro degradation buffer has a physiological pH value and is stored at 37 °C, in this study no enzymes were added which would make a significant difference to the conditions in vivo, especially for enzyme-degradable polymers. Since PLGA is prevailingly degraded by chemical hydrolysis, the difference between in vitro and in vivo degradation times should not be significant, but can still not be neglected [33,38]. For instance, Wang et al. could demonstrate a faster degradation of PLGA (75/25) in vivo [52] and Cai et al. showed that trypsin can accelerate the degradation rate of PLGA in vitro [53].

As mentioned earlier, different tissues require a varying degradation time of the implant to support the specific tissue regeneration [33,48]. For a successful implantation, besides the suitable degradation time, the surface including the binding sites for cells should not change adversely during the degradation process to avoid negative effects on the de novo cell adhesion [54]. Several studies investigated the in vivo biocompatibility and degradation of resorbable scaffolds [55,56,57]. A limited number of publications studied the in vitro or in vivo biocompatibility of in vitro degraded resorbable polymer constructs [58]. In our in vitro study under physiological conditions, the progressing degradation and the resulting smoother surface did not affect the adhesion and proliferation of hAD-MSCs. The cells were able to grow on the degraded constructs as well as on the non-degraded constructs and showed even a slightly higher cell viability after seven days of cultivation compared to cells cultured on the cell culture surface. Since no morphological changes of the cells on the degraded constructs were detected either, the RESOMER^®^ Filament LG D1.75 can be considered biocompatible also after degradation [29,39]. Therefore, it can be assumed that even after implantation into the human body and the subsequent degradation of LG D1.75, cell growths on constructs is possible and no detachment from the surface will appear over time. Nevertheless, in vivo experiments would be necessary to investigate inflammatory reactions of the body. Moreover, mechanical properties of degraded 3D printed construct must be studied in the future.

RESOMER^®^ Filament LG D1.75 could be a valuable material for 3D printed, resorbable bone implants in regenerative medicine and tissue engineering. On the one hand, it can replace missing bone areas as an accurately fitting implant by supporting the patient’s own bone cells during regeneration [54,59]. On the other hand, constructs printed with LG D1.75 could be colonized with the patient’s own cells before autologous transplantation, so that recovery and new bone formation can be additionally supported [49]. For such applications of LG D1.75 it was important to test whether the osteogenesis of human cells on the constructs can be inhibited by the material properties and components. In addition to the material, there is increasing interest in the search for xeno-free alternatives for FCS as media supplementation in regenerative medicine. hPL could provide such an alternative since the use of hPL was already shown to improve proliferation and differentiation in 2D and 3D cultivations of MSCs [40,41,42,60,61]. hPL contains many bioactive factors, which act synergistically to promote the attachment, proliferation, and differentiation of MSCs [40,41,42,60,61]. Most of the recent studies of PLGA as resorbable implant material were still performed with FCS as a medium supplement for cell culture [46,47]. For clinical applications, however, it is crucial to be able to replace FCS with an xeno-free alternative to avoid xenogeneic immune reactions and the transmission of prions or other zoonotic infections after transplantation [62,63,64].

In this study, we investigated the influence of FCS, HS, and hPL on the cell morphology and osteogenic differentiation on printed LG D1.75. As described already in a previous study, the hAD-MSCs in all three tested supplements were migrating on the construct surface along the printing direction [15]. The hAD-MSCs colonized on printed RESOMER^®^ Filament LG D1.75 showed osteogenic differentiation after three weeks in medium supplemented with FCS, HS, and hPL. As described earlier in several studies, in comparison to HS and FCS, hPL showed an accelerated and improved osteogenic differentiation of hAD-MSCs [40,41,42]. Altaie et al. described an osteoconductive influence of hPL after coating scaffolds with hPL [61]. Additionally, to the osteoconductive effect of hPL, several studies described also an osteoconductive influence of primarily porous PLGA scaffolds [46,65,66,67,68]. In this study, the differentiation behavior of the cells on LG D1.75 was more strongly supported by hPL than on the cell culture surface. Thus, the bulk construct of LG D1.75 can be described as osteoconductive in its function as a scaffold, but not as osteoinductive because of the lack of stimulation for osteogenesis [46,54,59,65,66,67,68]. Although histological staining with BCIP/NBT and alizarin red revealed higher osteogenic differentiation degree in terms of ALP-positive cells and calcium accumulation by cells in the presence of hPL, a deeper differentiation analysis must be performed in future experiments. Gene expression of osteogenic markers and immunofluorescent detection of specific proteins will give a closer insight into the cellular behavior in the presence of different supplements. However, the obtained results display the advantage of the combination of hPL supplemented cells cultivated on 3D printed PLGA constructs for bone tissue engineering.

## 5. Conclusions

We demonstrated a possible application of RESOMER^®^ Filament LG D1.75 as a material for fabrication of 3D printed, resorbable bone implants. Our in vitro experiments indicate that no cytotoxic effects can be expected after implantation. No toxic leachables and no inhibition of cell growth directly on the constructs were detected. The combination of xeno-free cultivation conditions with hPL and RESOMER^®^ Filament LG D1.75 as 3D printed scaffold supported the osteogenic differentiation of hAD-MSCs. RESOMER^®^ Filament LG D1.75 is suitable as a material for resorbable, 3D printed bone implants because of its remaining biocompatibility after degradation.

Taken together, the suitability of the material RESOMER^®^ Filament LG D1.75 to produce 3D printed, resorbable implants was demonstrated. Furthermore, the favorable utilization of hPL for xeno-free cultivation of human MSCs on such implants for possible autologous implantation was shown. The possibility to use hPL as xeno-free medium supplement for cell cultivation on biocompatible resorbable 3D printed scaffolds represents a promising sustainable combination with a great potential for future applications in regenerative medicine.

## Supplementary Materials

The following are available online at https://www.mdpi.com/1996-1944/13/15/3399/s1, Figure S1: Representative picture of the determination of the average filament size of 3D printed constructs. Scale bar 100 µm. Figure S2: Micrographs of constructs after 0, 3, 4, 5, and 6 months of degradation. A: 500× magnification of the construct surface in 2D and 3D taken with 3D microscope. Scale bar 100 µm. B: 3000× magnification of the construct surface. Scale bar 25 µm. Figure S3: Morphological examination of hAD-MSCs growing on degraded printed RESOMER^®^ Filament LG D1.75 (0, 3, 4, 5, and 6 months). After cultivation of 1, 3, and 7 days, the cells were stained with calcein-AM; 4× objective, scale bar 100 µm. Figure S4: Osteogenic differentiation of hAD-MSCs cultivated on the cell culture surface and stained with alizarin red 7, 14, and 21 days after induction of the osteogenic differentiation under the influence of FCS, HS, and hPL. Proliferation control after week 3; 4× objective, scale bar 200 µm. Figure S5: Osteogenic differentiation of hAD-MSCs cultivated on printed RESOMER^®^ Filament LG D1.75 and stained with alizarin red seven days after induction of the osteogenic differentiation under influence of FCS, HS and hPL; 4× objective, scale bar 200 µm. Figure S6: Alkaline phosphatase staining of differentiated hAD-MSCs cultivated and for seven days differentiated on printed RESOMER^®^ Filament LG D1.75. Scale bar 2500 µm.
