# Supplementary material for: Xeno-Free In Vitro Cultivation and Osteogenic Differentiation of hAD-MSCs on Resorbable 3D Printed RESOMER®
Source: Materials (Basel). 2020 Jul 31;13(15):3399. doi: 10.3390/ma13153399 (PMC7436127; doi:10.3390/ma13153399)
Supplement: Supplementary file 1 [file materials-13-03399-s001.pdf]

materials

Supplementary Materials: Xeno-Free In Vitro Cultivation and Osteogenic Differentiation of hAD-MSCs on Resorbable 3D Printed RESOMER®

Marline Kirsch [1], Annabelle-Christin Herder [1], Cécile Boudot [2], Andreas Karau [2], Jessica Rach [3], Wiebke Handke [4], Axel Seltsam [4], Thomas Scheper [1] and Antonina Lavrentieva [1,*]

[1] Institute of Technical Chemistry, Leibniz University Hannover, Callinstraße 5, 30167 Hannover, Germany; kirsch@iftc.uni-hannover.de (M.K.), annabelle.herder@gmail.com (A.-C.H.), scheper@iftc.uni-hannover.de (T.S.)

[2] Evonik Nutrition & Care GmbH, Business Line Health Care, Kirschenallee, 64293 Darmstadt, Germany; cecile.boudot@evonik.com (C.B.), andreas.karau@evonik.com (A.K.)

[3] German Red Cross Blood Service NSTOB, Institute Springe, Eldagsener Straße 38, 31830 Springe, Germany; Jessica.Rach@bsd-nstob.de

[4] Bavarian Red Cross Blood Service, Institute Nuremberg, Heimerichstrasse 57, 90419 Nuremberg, Germany; w.handke@blutspendedienst.com (W.H.), a.seltsam@blutspendedienst.com (A.S.)

* Correspondence: lavrentieva@iftc.uni-hannover.de; Tel.: +49-511-762-2955

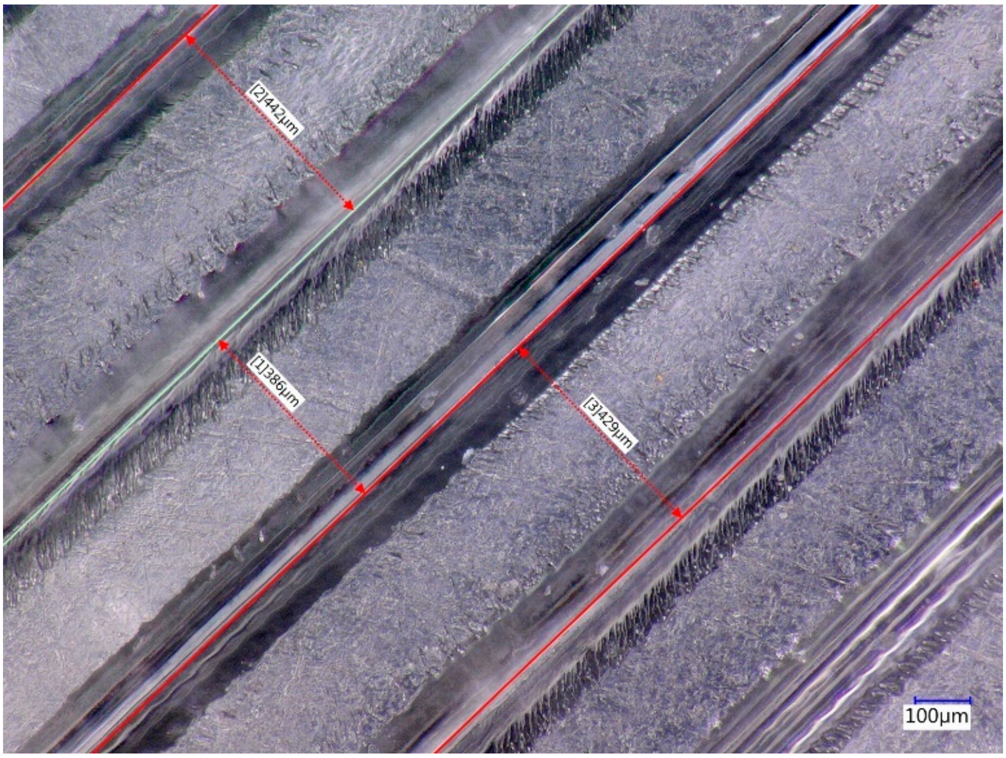

Figure S1. Representative picture of the determination of the average filament size of 3D printed constructs. Scale bar 100 µm.

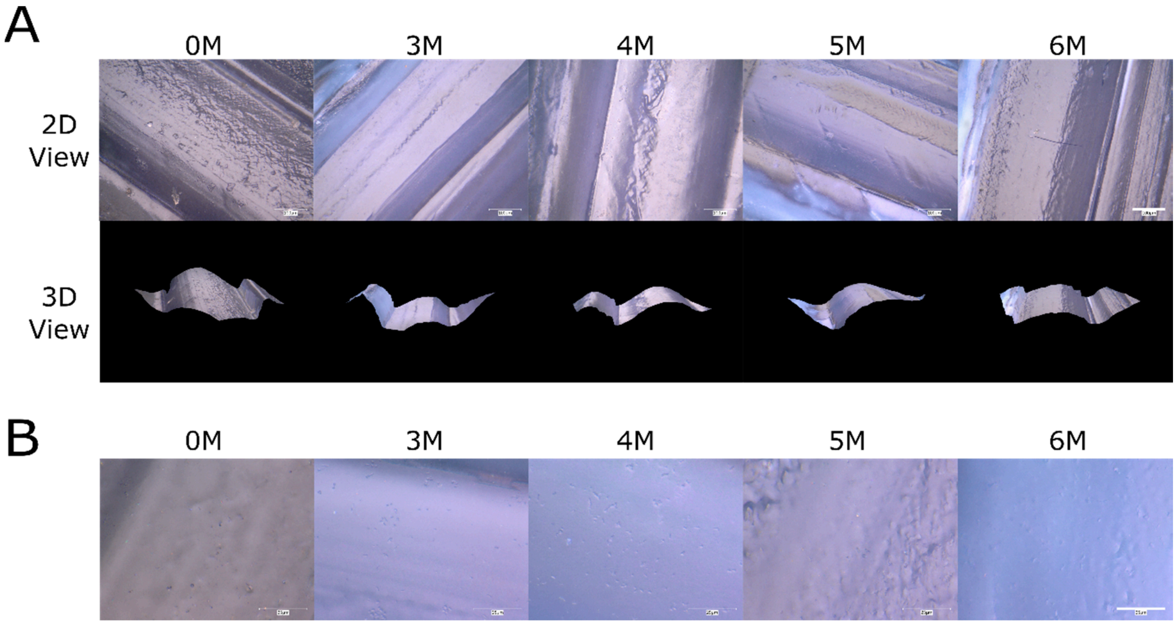

Figure S2. Micrographs of constructs after 0, 3, 4, 5 and 6 months of degradation. (**A**) 500 × magnification of the construct surface in 2D and 3D taken with 3D microscope. Scale bar 100 µm. (**B**) 3000 × magnification of the construct surface. Scale bar 25 µm.

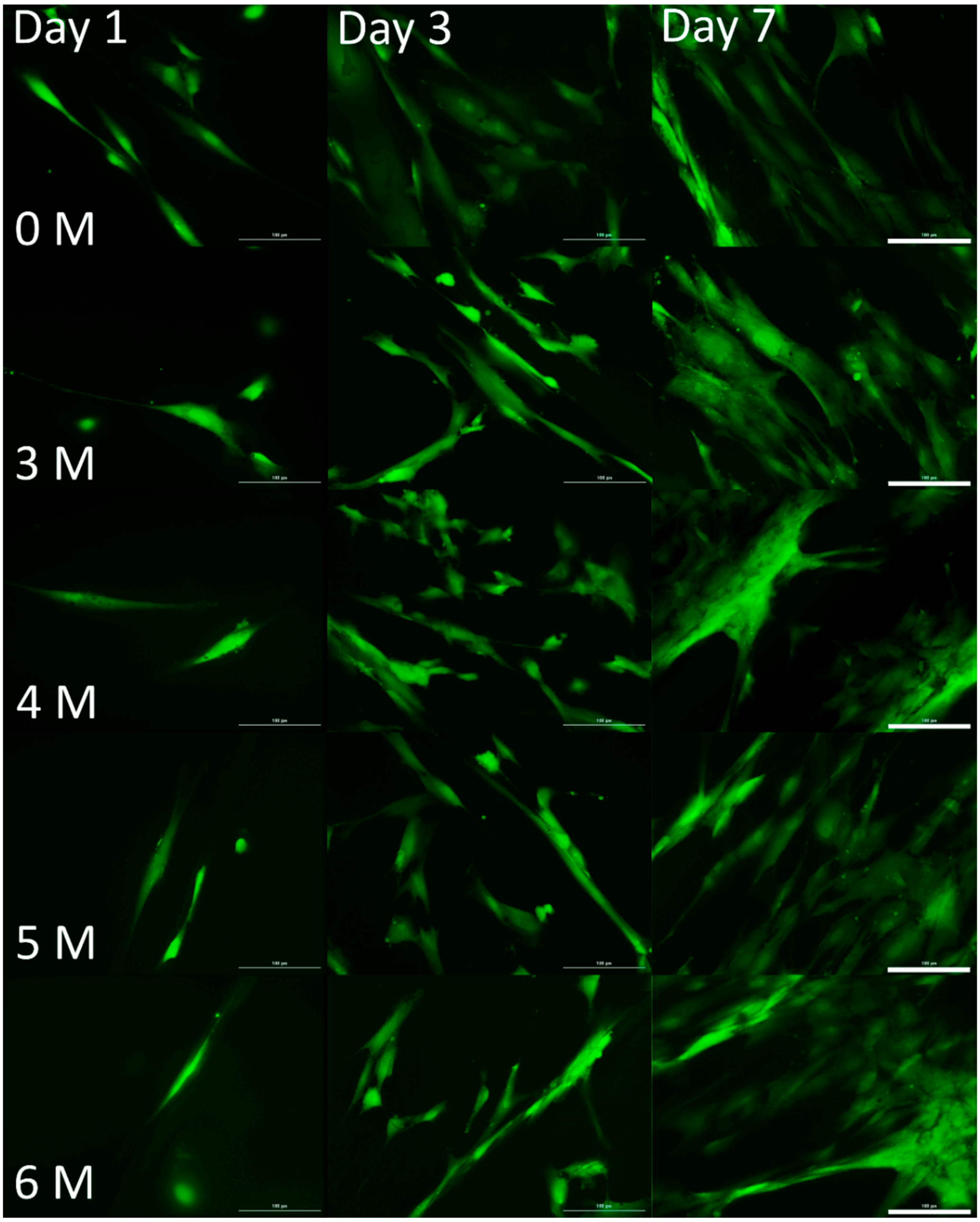

Figure S3. Morphological examination of hAD-MSCs growing on degraded printed RESOMER® Filament LG D1.75 (0, 3, 4, 5, and 6 months). After cultivation of 1, 3, and 7 days, the cells were stained with calcein-AM; 4× objective, scale bar 100 µm.

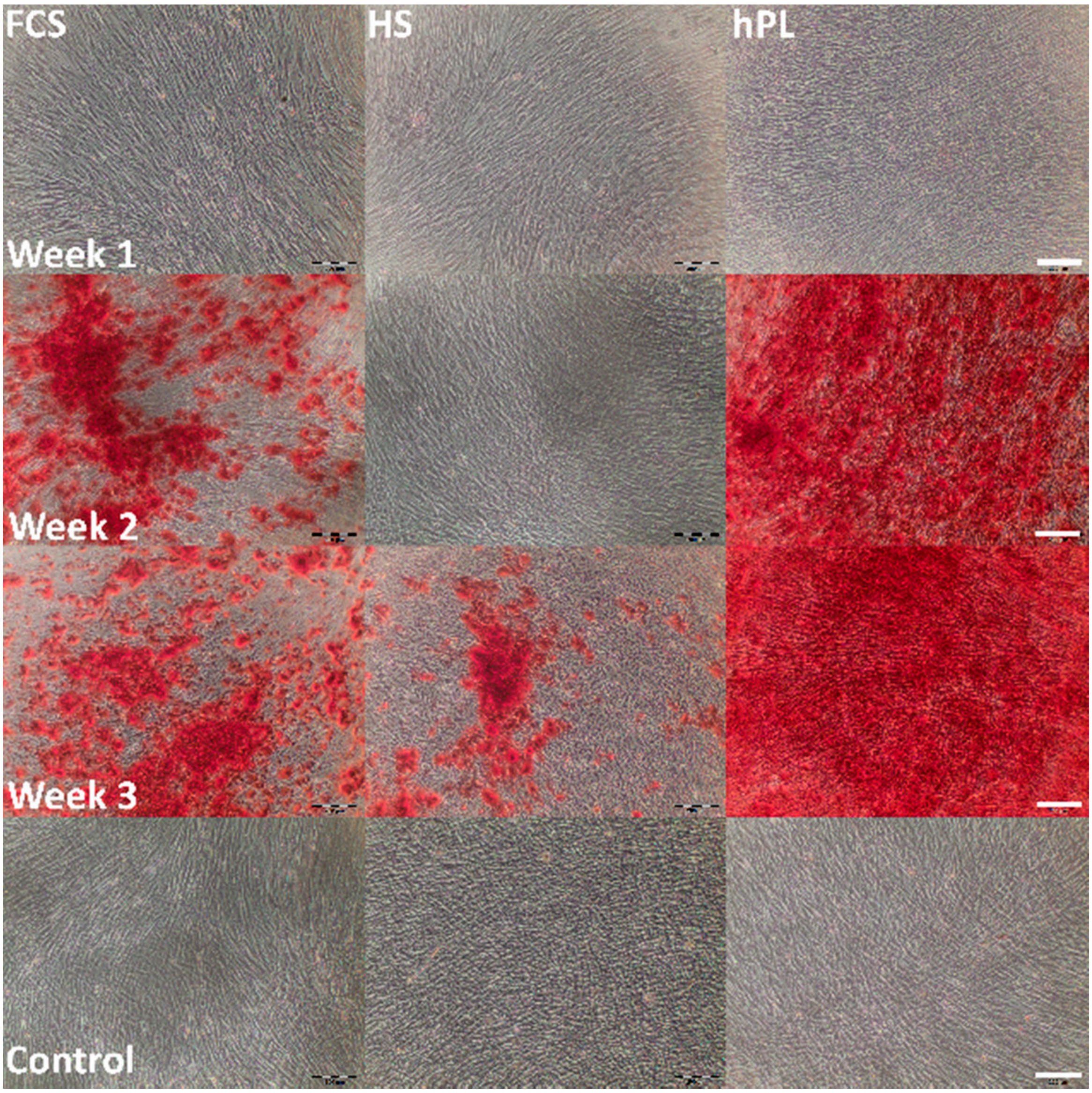

Figure S4. Osteogenic differentiation of hAD-MSCs cultivated on the cell culture surface and stained with Alizarin Red 7, 14 and 21 days after induction of the osteogenic differentiation under influence of FCS, HS and hPL. Proliferation control after week 3; 4 × objective, scale bar 200 μm.

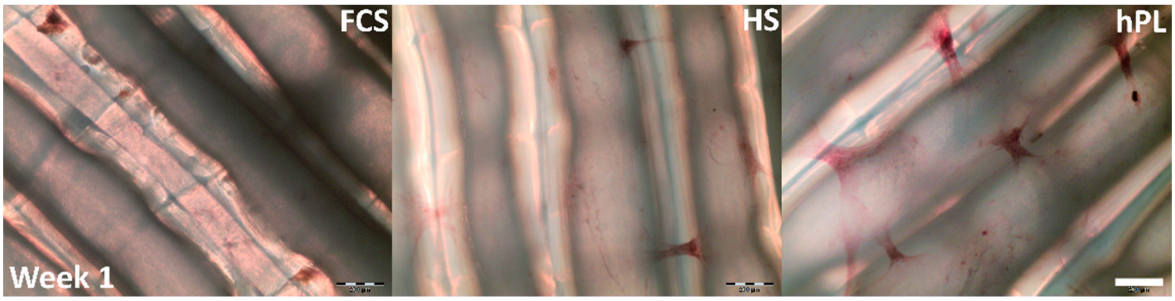

Figure S5. Osteogenic differentiation of hAD-MSCs cultivated on printed RESOMER® Filament LG D1.75 and stained with Alizarin Red seven days after induction of the osteogenic differentiation under influence of FCS, HS and hPL; 4 × objective, scale bar 200 μm.

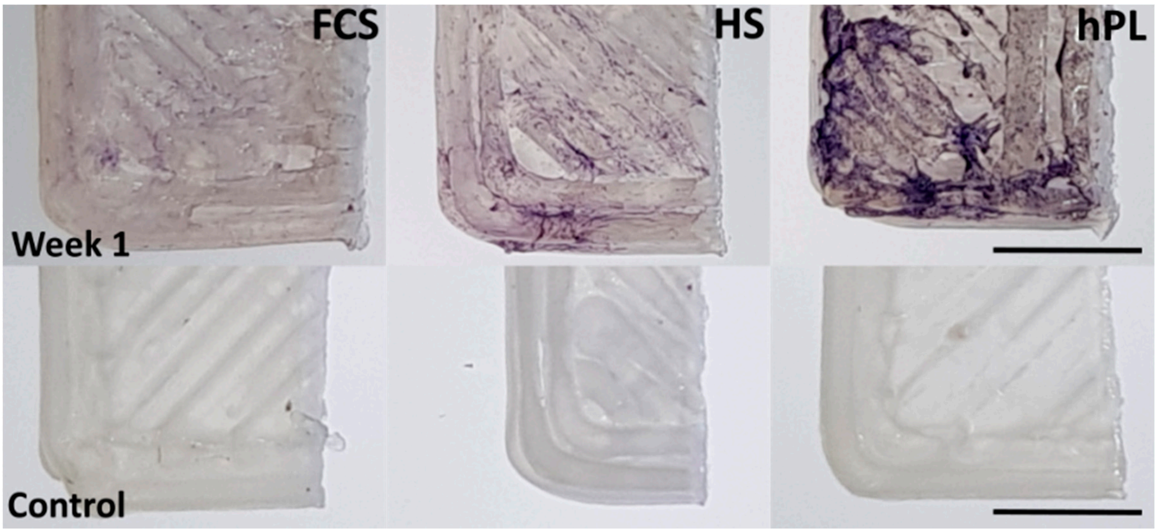

Figure S6. Alkaline phosphatase staining of differentiated hAD-MSCs cultivated and for seven days differentiated on printed RESOMER® Filament LG D1.75. Scale bar 2500 μm.

© 2020 by the authors. Submitted for possible open access publication under the terms and conditions of the Creative Commons Attribution (CC BY) license (http://creativecommons.org/licenses/by/4.0/).
